# Oral *Candida* colonisation and cariogenic bacteria in children and adolescents with cleft lip and/or palate: A systematic review and meta-analysis

**DOI:** 10.1016/j.jobcr.2026.101555

**Published:** 2026-10-09

**Authors:** Muhammad Irpan Hendrawan, Elsa Ayu Amanda, Ita Purnama Alwi, Husni Mubarak, Irham Taufiqurrahman, Andi Tajrin

**Affiliations:** a Department of Oral and Maxillofacial Surgery, Faculty of Dentistry, Hasanuddin University, Makassar, Indonesia; b Department of Oral and Maxillofacial Surgery, Faculty of Dentistry, Muhammadiyah University of Palangka Raya, Palangka Raya, Indonesia; c Department of Orthodontics and Dentocraniofacial Orthopedics, Faculty of Dentistry, Hasanuddin University, Makassar, Indonesia; d Department of Orthodontics and Dentocraniofacial Orthopedics, Faculty of Dentistry, Muhammadiyah University of Palangka Raya, Palangka Raya, Indonesia; e Department of Oral and Maxillofacial Surgery, Faculty of Dentistry, Lambung Mangkurat University, Banjarmasin, Indonesia; f Hasanuddin University Dental Hospital, Makassar, Indonesia

**Keywords:** Cleft lip and palate, Oral *Candida*, *Streptococcus mutans*, Dental caries, Oral microbiota, Sustainable development goals

## Abstract

**Purpose:**

Children with cleft lip and/or palate (CL/P) face a disproportionate cariogenic burden, yet the role of oral *Candida* and its partnership with cariogenic bacteria remains poorly quantified. This systematic review and meta-analysis synthesised observational evidence on oral *Candida* colonisation, cariogenic bacterial carriage, and clinical caries outcomes in non-syndromic CL/P, with GRADE-rated certainty.

**Methods:**

Following PRISMA 2020 and the Cochrane Handbook (PROSPERO CRD420261402624), six databases were searched from inception to 27 May 2026. Eligible studies were observational designs enrolling children aged 0–18 years with non-syndromic CL/P versus non-cleft controls. Risk of bias used ROBINS-E. Random-effects meta-analyses (DerSimonian–Laird) pooled risk ratios (RR) for dichotomous outcomes and standardised mean differences (SMD) for continuous caries indices, with GRADE certainty.

**Results:**

Ten studies (2011–2026; 421 CL/P vs 567 controls) were included. Oral *Candida* carriage was nearly threefold higher in CL/P (RR 2.94, 95% CI 2.04–4.22; *I*^2^ = 23.4%; *k* = 4), with *C. albicans* predominant. High salivary *Streptococcus mutans* counts were similarly elevated (RR 2.74, 95% CI 1.42–5.27; *I*^2^ = 0%), whereas *Lactobacillus* carriage showed no significant difference (RR 1.33, 95% CI 0.71–2.47). Caries indices were directionally higher in three of four datasets but highly heterogeneous (SMD 0.74; *I*^2^ = 92.3%). GRADE certainty was Low to Very Low.

**Conclusion:**

CL/P children carry elevated oral *Candida* and *S. mutans* burdens, supporting an early fungal–bacterial cariogenic shift. *Candida* assessment should be integrated into caries-risk evaluation from the deciduous-dentition stage within the multidisciplinary cleft pathway.

## 1 Introduction

Orofacial clefts (OFCs) are among the most common congenital craniofacial anomalies, and although global incidence and mortality fell between 1990 and 2021, prevalent cases continue to accumulate and are projected to rise through 2050.^1^ The burden is unevenly distributed, with over 80% of prevalent cases and 96% of OFC-related deaths in low- and middle-income countries.^2^ Indonesia illustrates this gap, where children wait until a mean age of 4.3 years for primary labioplasty.^3^

Cleft lip and/or palate (CL/P), the commonest OFC subtype, carries a cariogenic burden that is structural, salivary and microbial. Dental anomalies cluster in the cleft region, including tooth agenesis, microdontia, supernumerary teeth and enamel hypoplasia, forming plaque-retentive niches that invite demineralisation.^4^ Reduced salivary flow and peri-cleft biofilm dysregulation erode enamel even when hygiene is adequate, and caries experience is higher across both dentitions, widening in lower-resource settings.^5^ Raised *Streptococcus mutans* colonisation and fungal–bacterial cross-talk drive an acidogenic shift that sets CL/P apart from peers.^6^

Meta-analysis has steadily sized this burden: across 20 observational studies, affected children showed more caries (SMD 0.46, 95% CI 0.15–0.77), peaking in mixed dentition.^7^ What remains missing is a mechanistic account of the cariogenic ecology, above all the role of *Candida* and its partnership with *S. mutans*.

*Candida* has emerged as a keystone fungal partner, with paediatric oral colonisation of 10–70% that routinely exceeds non-cleft peers.^8^ The residual palatal defect and acrylic appliances such as obturator plates create a low-clearance, plaque-retentive niche favouring fungal persistence; colonisation is already near-universal in infants fitted with acrylic plates and far above controls.^9^ Mechanistically, *S. mutans*-derived glucosyltransferase B anchors the bacterium to *C. albicans* and, with quorum signalling, yields a polymicrobial biofilm more acid-tolerant and cariogenic than either species alone.^10^ Non-*albicans* species, especially *C. tropicalis*, can rival *C. albicans* in this partnership, echoing the mix seen in cleft infants.^11^

Against this background, we synthesised observational evidence on oral *Candida* colonisation, cariogenic bacterial burden and clinical caries outcomes in children and adolescents with non-syndromic CL/P, deriving pooled estimates with GRADE-rated certainty. We hypothesised that, beyond raised *S. mutans* carriage, *Candida* colonisation and DMFT/dmft burden would be higher in CL/P than in non-cleft peers, varying by dentition stage and care context.

## 2 Methods

### 2.1 Protocol and registration

This systematic review and meta-analysis was reported in line with the PRISMA 2020 statement and conducted following the Cochrane Handbook for Systematic Reviews of Interventions (version 6.5).^12^^,^^13^ The protocol was prospectively registered in PROSPERO (CRD420261402624) before screening, with no subsequent amendments.^14^

### 2.2 Eligibility criteria

Eligibility was defined a priori using the PECO framework (Population, Exposure, Comparator, Outcome), with study design specified separately and full criteria summarised in Table 1. In brief, we included observational primary studies of children and adolescents aged 0–18 years with non-syndromic cleft lip and/or palate (CL/P).

**Table 1:** Eligibility criteria based on the PECO framework with study design.

Domain	Inclusion	Exclusion
Population (P)	Children and adolescents aged 0–18 years	Adults (≥19 years); mixed populations without pediatric subgroup data
Exposure (E)	Nonsyndromic cleft lip with or without cleft palate (CL/P), encompassing cleft lip only (CLO), cleft palate only (CPO), and combined cleft lip and palate (CLP); unilateral or bilateral	Syndromic clefts (e.g., Van der Woude syndrome, Stickler syndrome, 22q11.2 deletion syndrome); cleft-like lesions of non-developmental origin
Comparator (C)	Healthy non-cleft children and adolescents (primary); within-CL/P comparisons stratified by caries status (caries-free vs. high-caries) as secondary	None additional
Outcome (O)	(i) Quantitative oral microbiological burden of cariogenic taxa — *Streptococcus mutans*, *Lactobacillus*, and *Candida* spp. — assessed by quantitative culture (CFU), qPCR, or sequencing-based approaches (16S rRNA, ITS, or shotgun metagenomics); and/or (ii) clinical caries outcomes (dmft/DMFT, dmfs/DMFS, deft, ICDAS, Caries Severity Index [CSI], or caries prevalence as a binary outcome)	Studies with insufficient outcome data for extraction after attempted contact with the corresponding author(s)
Study design	Original observational primary studies: cross-sectional, case–control, prospective or retrospective cohort	Case reports or case series with <5 participants per group; conference abstracts, editorials, letters, narrative reviews; animal, *in vitro*, or *ex vivo* studies

Abbreviations: CL/P = cleft lip and/or palate; CFU = colony-forming units; qPCR = quantitative polymerase chain reaction; ITS = internal transcribed spacer; ICDAS = International Caries Detection and Assessment System.

### 2.3 Information sources

Six electronic databases were searched from inception to 27 May 2026, with no language or date restrictions: PubMed/MEDLINE, Embase (via Ovid), Scopus, ScienceDirect, Web of Science Core Collection, and the Cochrane Library. Reference lists of all included studies and relevant reviews were also screened for eligible records.

### 2.4 Search strategy

The search combined controlled vocabulary (MeSH in PubMed and the Cochrane Library; Emtree in Embase) with free-text terms across three concept blocks: orofacial cleft phenotypes, cariogenic oral microbiota, and dental caries outcomes. Boolean operators, truncation, and database-specific field tags (e.g., TITLE-ABS-KEY in Scopus, /exp in Embase) were applied. Full search strings and per-database hits are given in Table 2.

**Table 2:** Search strategy for each database.

Database	Search strategies	Hits
**PubMed**	((“orofacial cleft*" OR “cleft lip” OR “cleft palate” OR “cleft lip and palate”) AND (“*Streptococcus mutans*” OR “Lactobacillus” OR “Candida” OR “cariogenic” OR “oral microbiota” OR “oral microbiome” OR “dental plaque”) AND (“dental caries” OR “tooth decay” OR “DMFT” OR “dmft” OR “ICDAS” OR “early childhood caries"))	23
**Embase**	(‘orofacial cleft'/exp OR ‘cleft lip'/exp OR ‘cleft palate'/exp OR ‘orofacial cleft*' OR ‘cleft lip’ OR ‘cleft palate’) AND (‘streptococcus mutans'/exp OR 'lactobacillus'/exp OR ‘candida'/exp OR ‘mouth flora'/exp OR ‘cariogenic’ OR ‘oral microbiota’ OR ‘dental plaque’) AND (‘dental caries'/exp OR ‘dental caries' OR ‘tooth decay’ OR ‘DMFT’ OR 'ICDAS' OR ‘early childhood caries')	25
**Cochrane Library**	#1: (“orofacial cleft” OR “orofacial clefts” OR “cleft lip” OR “cleft palate” OR “cleft lip and palate” OR “cleft lip with or without cleft palate")	6
	#2: [mh “Cleft Lip"] OR [mh “Cleft Palate"]	
	#3: (“*Streptococcus mutans*” OR “Lactobacillus” OR “Candida” OR “cariogenic” OR “oral microbiota” OR “oral microbiome” OR “dental plaque")	
	#4: [mh “*Streptococcus mutans*"] OR [mh “Lactobacillus"] OR [mh “Candida"] OR [mh “Microbiota"] OR [mh “Dental Plaque"]	
	#5: (“dental caries” OR “tooth decay” OR “DMFT” OR “dmft” OR “ICDAS” OR “early childhood caries")	
	#6: [mh “Dental Caries"]	
	#7: #1 OR #2	
	#8: #3 OR #4	
	#9: #5 OR #6	
	#10: #7 AND #8 AND #9	
**Scopus**	(TITLE-ABS-KEY(“orofacial cleft*" OR “cleft lip” OR “cleft palate” OR “cleft lip and palate” OR “cleft lip with or without cleft palate")) AND (TITLE-ABS-KEY(“*Streptococcus mutans*” OR “Lactobacillus” OR “Candida” OR “cariogenic” OR “oral microbiota” OR “oral microbiome” OR “dental plaque")) AND (TITLE-ABS-KEY(“dental caries” OR “tooth decay” OR “DMFT” OR “dmft” OR “ICDAS” OR “early childhood caries"))	24
**ScienceDirect**	“cleft lip and palate” AND (“*Streptococcus mutans*” OR “*Candida albicans*”) AND “dental caries” AND (“caries prevalence” OR “caries experience")	21
**Web of Science**	(“cleft lip” OR “cleft palate”) AND (“*Streptococcus mutans*” OR “Candida”) AND (“dental caries” OR “DMFT”) AND (“child” OR “children” OR “pediatric” OR “paediatric")	66

### 2.5 Selection process

Records were imported into Mendeley Reference Manager for automatic de-duplication (by DOI and title), with residual duplicates removed manually in Excel. Two reviewers (M.I.H. and E.A.A.) independently screened titles and abstracts, and eligible records proceeded to independent full-text assessment. A third reviewer (I.T.) was pre-designated as adjudicator but not required, as disagreements were resolved by discussion. Inter-rater agreement was not formally quantified, a limitation acknowledged below. Exclusion reasons were recorded in the PRISMA 2020 flow diagram (Fig. 1).

**Fig. 1:**
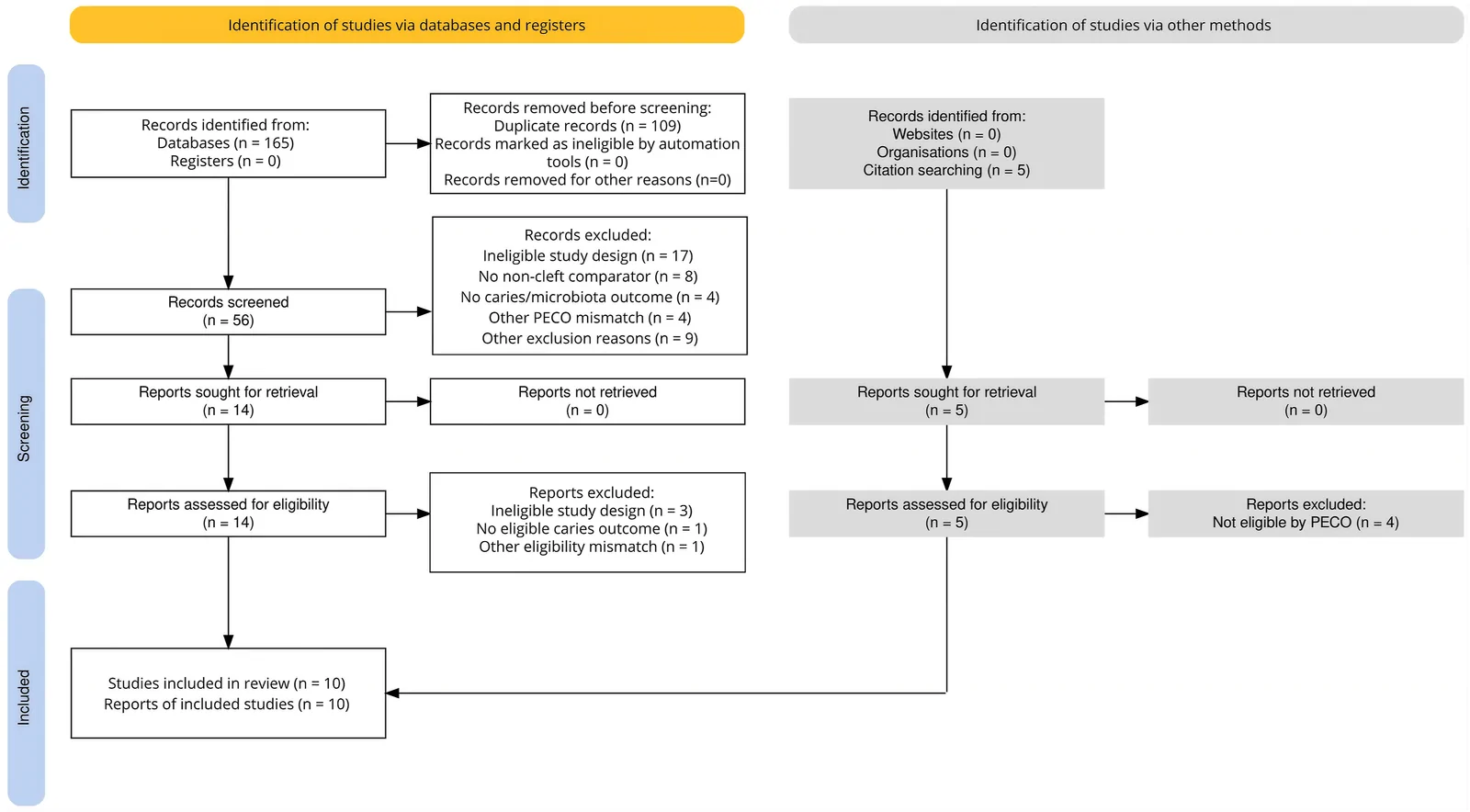
PRISMA 2020 flow diagram of study selection.

### 2.6 Data collection process and data items

Two reviewers (M.I.H. and E.A.A.) extracted data independently using a standardised Microsoft Excel form piloted on two studies, with discrepancies resolved by consensus after joint re-examination of the source publications. The following items were extracted:

• Study characteristics: first author, year, country and setting, design, and recruitment period;
• Participants: sample size per group, age, sex, cleft phenotype (CLO/CPO/CLP; unilateral or bilateral), surgical history (cheiloplasty, palatoplasty, alveolar bone grafting), orthodontic status, and comparator definition;
• Exposure and microbiological assessment: sampling site and technique, microbiological method (culture, qPCR, or sequencing), target organisms, quantification unit (CFU/mL, relative abundance, or categorical thresholds), and laboratory protocols;
• Outcomes: clinical caries indices (dmft/DMFT, dmfs/DMFS, deft, ICDAS, CSI), caries prevalence, microbial colonisation frequencies, and effect estimates with uncertainty;
• Confounders and effect modifiers: oral hygiene, diet, socioeconomic status, antibiotic and antifungal exposure, breastfeeding history, and use of pre- and post-surgical appliances (e.g., PNAM, obturator).

When data were missing, graphical only, or insufficient, corresponding authors were emailed with one reminder after two weeks; absent a reply within four weeks, only published data were used. Extracted data appear in Table 3, Table 4, Table 5.

**Table 3:** Characteristics of included studies.

Study	Country (Year)	Design	n CL/P/Control	Age range	Cleft phenotype	Surgical status at sampling	Key outcomes reported
Hassani et al.^15^	USA (2020)	Cross-sectional, PNAM-stratified	31 (23 PNAM + 8 no-PNAM)/30	0–18 mo	CLP (PNAM arm); mixed CL/CP/CLP (no-PNAM arm)	Pre-cheiloplasty	SM/LB CRT; CFU; salivary pH
Rawashdeh et al.^16^	Jordan (2011)	Cross-sectional, age-stratified (G1–G3)	60/60	0 mo – ≥17 y	UCLP + BCLP + CPO (pooled)	Variable, not stratified	*Candida* prevalence; DMFT; dmft
Shashni et al.^17^	India (2015)	Cross-sectional, 3-group (CL/P vs non-cleft high-caries-risk vs non-cleft caries-free)	23/25 (caries-free Group III; high-caries-risk Group II n = 25 excluded a priori — selection-biased reference)	4–9 y	CLP (subtypes unspecified)	Not stratified; not analytically reported	MS (Dentocult-SM), LB (Rogosa MRS agar); deft + DMFT; Snyder caries-activity
Durhan et al.^18^	Turkey (2019)	Longitudinal cohort (24 mo; 3 timepoints)	21/13	0–30 mo	UCLP + BCLP + CP-only mixed	Cheiloplasty + palatoplasty during follow-up	Caries prevalence; MS; LB; *Candida*
Funahashi et al.^19^	Japan (2019)	Cross-sectional, metatranscriptomic	6/4	7–15 y	UCLP + BCLP mixed	Post-surgical, mixed	DMFT; 16S + RNA-seq
Sundell et al.^20^	Sweden (2015)	Population-based registry cross-sectional	133/297	5 and 10 y (two cohorts)	UCLP + BCLP + CP (all)	Variable	MS, LB (Cariogram); OH; caries
Khan et al.^21^	India (2023)	Cross-sectional, case–control	50/50	1–14 y	Alveolus 25, palate 4, soft palate 6, UCLP 12, BCLP 3	Variable, not stratified	*Candida* CFU; speciation
Sravani et al.^22^	India (2026)	Cross-sectional, infant cohort	40/40	0–3 y	CL/P (pooled)	Post-cheiloplasty	*Candida* prevalence + speciation; def; CSI
Zhang et al.^23^	China (2024)	Cross-sectional, 6-group (3 ABG status × 2 age strata)	87 (multi-group; healthy comparators within design)	12–30 y (adolescents + young adults)	AC (UCLP + BCLP)	ABG status: pre/peri/post-bone graft	16S V3–V4 supragingival plaque microbiome
Shi et al.^24^	China (2026)	Cross-sectional, 4-group (cleft × caries severity 2×2)	57 CLP (24 HC + 33 LC)/48 NCLP (22 HC + 26 LC)	6–12 y	UCLP + BCLP	Post-surgical, mixed	16S V3–V4 saliva; MDI; LEfSe; network analysis

**Table 4:** Oral sampling, microbiological methods, and caries measures across included studies.

Study	Oral sample/site	Microbiology method	Target organism(s)/scope	Output (unit)	Caries assessment	Caries outcome type	Synthesis note
Durhan et al.^18^	Infant intraoral swabs (serial); maternal saliva	MSB agar (MS); Rogosa agar (LB); Sabouraud (yeasts)	Mutans streptococci, lactobacilli, yeasts, *S. aureus*	CFU/mL (counts)	Visual-tactile; initial caries present/absent	Binary (caries yes/no)	Eligible for RR/OR on caries presence
Sundell et al.^20^	Stimulated whole saliva	Chair-side culture tests	Mutans streptococci; lactobacilli; buffer capacity; secretion	Category cut-offs	ICDAS-II	Continuous (dmfs/DMFS) + risk category	Risk-model primary; dmfs/DMFS may be extractable
Sravani et al.^22^	Multi-site oral swabs	Culture + speciation	*Candida albicans* vs non-*albicans*	CFU/mL + species category	def & CSI indices	Continuous	Strong candidate for MD meta (def/CSI)
Shi et al.^24^	Unstimulated saliva	16S rRNA sequencing (V3–V4)	Bacterial microbiome	Diversity + relative abundance	DMFS + dmfs strata; PLI/GI	Stratified	Narrative synthesis (omics heterogeneity)
Funahashi et al.^19^	Supragingival plaque	Metatranscriptomics	Active taxa (mRNA-to-rRNA ratio); networks	Activity ratios/network metrics	DMFT	Continuous	Narrative (omics; small n)
Zhang et al.^23^	Supragingival plaque pooled	16S rRNA sequencing + LEfSe/BugBase	Microbiome + predicted phenotypes	Relative abundance	DMFS/dmfs; PLI	Continuous	Narrative for microbiome; DMFS/dmfs potentially extractable
Rawashdeh et al.^16^	Tongue + buccal + palatal swabs	Culture + germ tube + VITEK	*Candida* spp.	Colonisation/species	WHO criteria	Continuous (DMFT/dmft)	*Candida* carriage may be meta-analysed if 2×2 extractable
Shashni et al.^17^	Stimulated saliva	Dentocult SM + Rogosa MRS agar (LB) + Snyder test	Mutans streptococci; lactobacilli; acidogenicity	Categories/CFU	Clinical exam	Continuous (deft/DMFT)	Meta possible if mean ± SD per group available
Khan et al.^21^	Tongue + buccal + palatal + floor of mouth swabs	Culture + speciation (germ tube + CHROMagar)	*Candida albicans* and non-*albicansCandida*	CFU (carriage rate)	WHO criteria (DMFT/dmft), PI, GI	*Candida* (primary); caries index reported overall	Use for *Candida* outcome with subset age G1 (≤5) + G2 (6–16); do not use DMFT/dmft for 0–18 synthesis (not age-stratified)
Hassani et al.^15^	Saliva	Selective culture + pH + ATP	Mutans streptococci; lactobacilli; acidity; ATP proxy	Colony counts + CRT/OHSU	Risk tests	Proxy outcome	Keep separate from clinical-caries meta

**Table 5:** Microbiological methods, thresholds, and caries assessment criteria.

Study	Microbiological method	“High count” threshold	Caries criteria	Examiner reliability (κ)	Sampling timing & specimen
Hassani et al.^15^	OHSU CRT (SM/LB); CFU plating	CRT ≥ 3 (≥10^5^ CFU/mL)	Not applicable (pre-eruption cohort)	NR	Morning, fasting; pooled oral swab
Rawashdeh et al.^16^	Sabouraud agar + germ-tube test	Any growth on Sabouraud	WHO 1997 (DMFT/dmft)	NR	Oral rinse + plaque swab
Shashni et al.^17^	Dentocult-SM Strip Mutans; Rogosa MRS agar (LB)	SM > 10⁵ CFU/mL (Score ≥2); LB detectable (≥10⁴ CFU/mL)	Modified Koch's criteria (deft + DMFT)	NR	Paraffin-stimulated saliva
Durhan et al.^18^	MSB agar (MS); Rogosa agar (LB); Sabouraud (yeasts)	SM/LB ≥ 10^5^ CFU/mL	Visual-tactile; initial caries present/absent	NR	Intraoral swabs at 3 timepoints (birth/first tooth/primary molar)
Funahashi et al.^19^	Metatranscriptomic total RNA-seq (Illumina MiSeq, 300-bp PE); 16S rRNA + mRNA reads	Not applicable (relative abundance)	WHO modified (DMFT)	NR	Supragingival plaque, pooled
Sundell et al.^20^	Dentocult-SM, Dentocult-LB; Cariogram v2.01	SM/LB ≥ 10^6^ CFU/mL	ICDAS	Standardised national protocol	Paraffin-stimulated saliva
Khan et al.^21^	Sabouraud + CHROMagar + germ-tube + sugar fermentation	≥ 10^2^ CFU/mL Sabouraud	WHO (DMFT/dmft)	NR	Oral rinse + tongue/palate swab
Sravani et al.^22^	Sabouraud + germ-tube; CHROMagar for speciation	> 10^2^ CFU/mL Sabouraud	WHO modified (def + CSI)	NR	Morning, fasting ≥ 1 h; oral swab + saliva
Zhang et al.^23^	16S rRNA (V3–V4) Illumina + LEfSe + PICRUSt	Not applicable (relative abundance)	DMFS/dmfs (criteria NR)	Not applicable	Supragingival plaque, pooled
Shi et al.^24^	16S rRNA (V3–V4) Illumina + LEfSe + MDI + network analysis	MDI > 8 for HC; DMFS + dmfs > 8 (HC)	WHO (DMFS + dmfs)	NR	Paraffin-stimulated whole saliva

### 2.7 Risk of bias assessment

The two reviewers (M.I.H. and E.A.A.) independently appraised each study with the Risk Of Bias In Non-randomised Studies of Exposure (ROBINS-E) tool, which suits observational exposure studies rather than intervention trials. Its seven domains (confounding, exposure measurement, participant selection, post-exposure interventions, missing data, outcome measurement, and selection of the reported result) were each rated low risk, some concerns, high risk, or very high risk, with overall judgements from the ROBINS-E signalling-question algorithm.^25^ Discrepancies were resolved by joint re-review until consensus, and visualisations were produced with the *robvis* application.^26^ No study was excluded for risk of bias; judgements instead informed interpretation and are reported in the Results and in Fig. 2A and B.

**Fig. 2:**
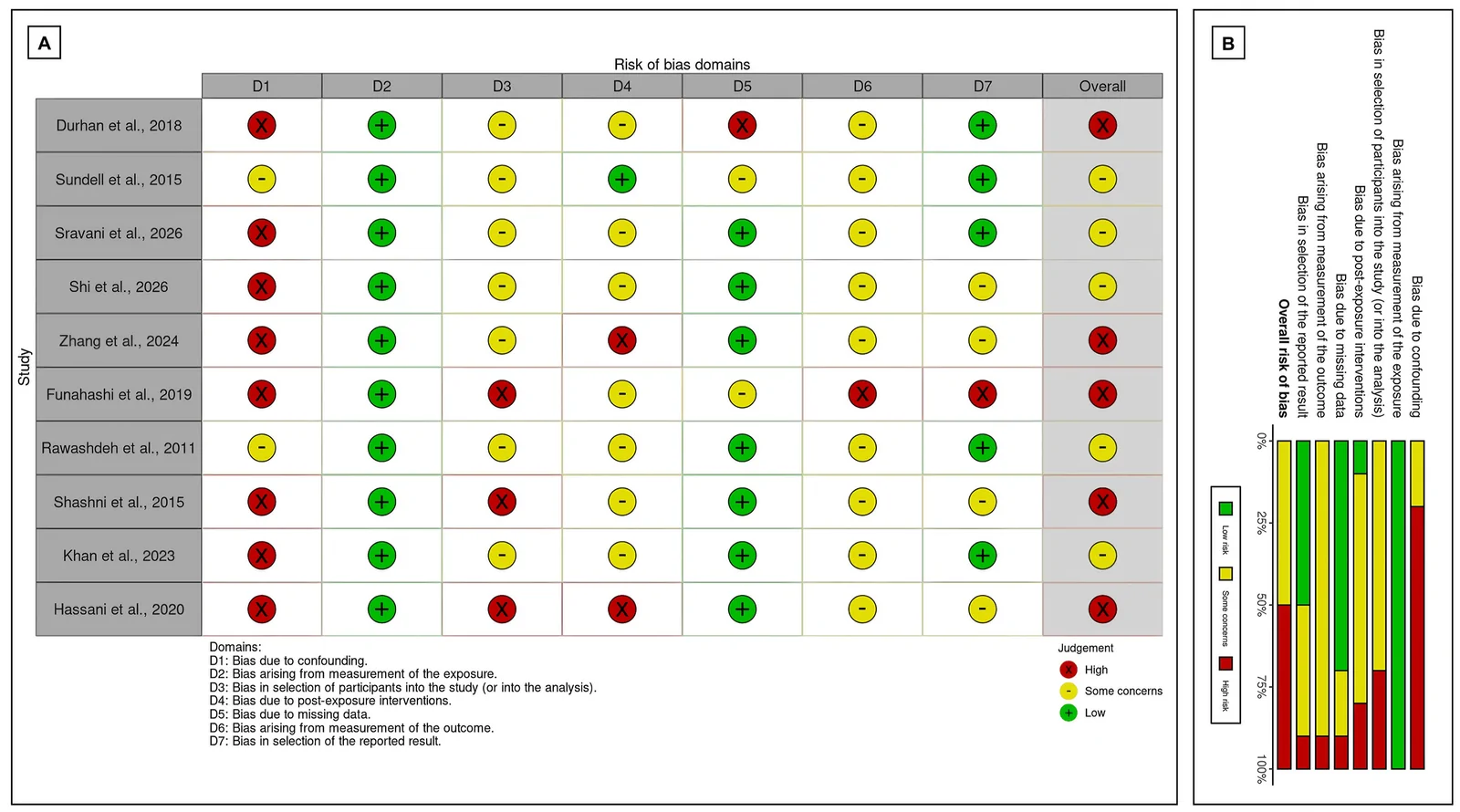
ROBINS-E assessment of the ten included studies. **(A)** Traffic-light plot of per-study and per-domain risk-of-bias judgements. **(B)** Weighted summary plot showing the proportional distribution of judgements across ROBINS-E domains.

### 2.8 Effect measures

Effect measures were pre-specified so that ratios above 1 or differences above 0 indicated higher burden in CL/P. For dichotomous outcomes (*Candida* colonisation, high salivary *S. mutans* and *Lactobacillus* spp. carriage, and caries prevalence) we used the risk ratio (RR) with 95% confidence interval (CI), preferred over the odds ratio because the outcomes were period prevalences rather than incident events; “high” carriage followed each study's own threshold. For continuous indices (dmft, DMFT, deft, CSI) we used the mean difference (MD) when indices matched and the standardised mean difference (SMD; Hedges' g) otherwise. Medians with interquartile ranges were to be converted via the method of Wan et al.^27^ and outcomes with fewer than two studies were synthesised narratively.

### 2.9 Synthesis methods

Pairwise random-effects meta-analyses were performed on Meta-Analysis Online (https://metaanalysisonline.com; accessed 30 May 2026), using the DerSimonian–Laird between-study variance estimator.^28^^,^^29^ A random-effects model was chosen a priori given the expected clinical and methodological heterogeneity across populations, phenotypes, techniques and count thresholds. Because this estimator can understate variance when few studies contribute, 95% prediction intervals were reported with every pooled estimate.^30^

Heterogeneity was quantified with the *I*^2^ statistic, interpreted per Section 10.10.2 of the Cochrane Handbook (low 0–40%, moderate 30–60%, substantial 50–90%, considerable 75–100%), alongside the between-study variance *τ*^2^ and Cochran's *Q*. Standardised mean differences were interpreted using Cohen's conventions of approximately 0.2 (small), 0.5 (medium) and 0.8 (large).^31^^,^^32^

Pooling proceeded only when an outcome was reported in at least three studies (*k* ≥ 3) with extractable group-level data (numerator/denominator or mean ± SD) and the contributing studies were judged sufficiently similar clinically and methodologically for interpretable pooling, per Section 10.10.4.1 of the Cochrane Handbook. Other outcomes were synthesised narratively, and forest plots were generated within the platform.

Two sensitivity analyses were pre-specified for each meta-analysed outcome: leave-one-out analysis to gauge the influence of individual studies, and restriction to studies at low or some-concerns overall risk of bias on ROBINS-E. Pre-specified subgroup analyses by cleft phenotype (CLO vs CPO vs CLP), age group (≤5, 6–12 and 13–18 years) and microbiological method (culture-based vs molecular) were contingent on sufficient stratified data.

### 2.10 Reporting bias assessment

Across-study reporting bias (publication bias and small-study effects) was pre-specified for evaluation by funnel-plot inspection and by formal asymmetry tests (Egger's regression and Begg's rank correlation), applied only when at least 10 studies contributed to a synthesis, following the recommended power threshold and Section 13.3.5.5 of the Cochrane Handbook.^13^^,^^33^ Funnel plots were inspected regardless of study count, with cautious interpretation where *k* < 10. Within-study selective reporting was addressed through Domain 7 of the ROBINS-E assessment.

### 2.11 Certainty assessment (GRADE)

The two reviewers independently rated the certainty of evidence for each meta-analysed outcome using the Grading of Recommendations, Assessment, Development and Evaluation (GRADE) framework, with disagreements resolved by consensus and guidance taken from the GRADE Handbook. Because all included studies were observational, the initial certainty was set at “low” per GRADE guidance for non-randomised evidence.^34^

Each outcome was assessed across five downgrading domains (risk of bias, inconsistency, indirectness, imprecision and publication bias) and three upgrading domains: large magnitude of effect (RR ≥ 2 or ≤ 0.5 with no plausible confounding fully explaining it), a dose–response gradient, and residual confounding that would bias the effect towards the null.

Final ratings were classified as High (⊕⊕⊕⊕), Moderate (⊕⊕⊕◯), Low (⊕⊕◯◯) or Very Low (⊕◯◯◯), and the full evidence profile is presented in the Summary of Findings (Table 6).

**Table 6:** Summary of findings (GRADE).

Outcome	k (participants)	Pooled effect (95% CI)	Absolute effect per 100 (95% CI)	Heterogeneity I^2^ (PI)	Certainty (GRADE)
*Candida* colonisation (MA-1)	4 (334)	RR **2.94 (2.04 to 4.22)**, P < 0.0001	Control 22 per 100 → CL/P 65 per 100; **43 more per 100 (23 more to 71 more)**	23.4% (PI 1.29 to 6.68)	⊕⊕◯◯ **Low**
*S. mutans* high count (MA-2)	3 (512)	RR **2.74 (1.42 to 5.27)**, P = 0.003	Control 4 per 100 → CL/P 10 per 100; **6 more per 100 (2 more to 15 more)**	0%	⊕◯◯◯ **Very Low**
*Lactobacillus* high count (MA-3)	3 (512)	RR **1.33 (0.71 to 2.47)**, P = 0.37	Control 5 per 100 → CL/P 6 per 100; **1 more per 100 (1 fewer to 7 more)**	0%	⊕◯◯◯ **Very Low**
DMFT/dmft/def burden (MA-4, narrative)	4 datasets (∼210)	SMD **0.74 (−0.55 to 2.03)**, P = 0.26	Narrative; directionally higher in CL/P in 3/4 datasets (excluding Funahashi,^19^ n = 6/4)	92.3%	⊕◯◯◯ **Very Low**
Caries prevalence (MA-5, single-study)	1 (34)	19.0% vs 0% at first incisor eruption (P = 0.144); 47.6% vs 38.5% at primary molar (P = 0.728)	19 more per 100 (first incisor); 9 more per 100 (primary molar) — single study, very imprecise	Not applicable	⊕◯◯◯ **Very Low**

## 3 Results

### 3.1 Study selection

Database searches retrieved 165 records across PubMed/MEDLINE, Embase, Scopus, ScienceDirect, Web of Science and Cochrane Library. After removing 109 duplicates, we screened 56 titles and abstracts against the a priori PECO criteria and excluded 42 (reasons detailed in Fig. 1). Of the 14 full texts assessed, 5 were excluded with reasons (ineligible design, n = 3; no eligible caries outcome, n = 1; other eligibility mismatch, n = 1), and citation searching added one further report. Ten studies thus entered the qualitative synthesis (4 in MA-1, *Candida* colonisation; 3 in MA-2, *Streptococcus mutans*; 3 in MA-3, *Lactobacillus* spp.), following PRISMA 2020 as shown in Fig. 1.^12^

### 3.2 Characteristics of included studies

The ten studies were published between 2011 and 2026. Across the nine with explicit two-group enrolment, 421 children and adolescents with CL/P were compared against 567 non-cleft controls, while Zhang et al.^23^ used a multi-group plaque-microbiome design with intra-study healthy comparators (Table 3). Origins were India (*k* = 3), China (*k* = 2), the USA, Jordan, Japan, Sweden and Turkey. Eight were cross-sectional or case–control studies; Durhan et al.^18^ was a 24-month longitudinal cohort with three timepoints, and Shashni et al.^17^ a three-group cross-sectional design. Ages ranged from 0 years (Sravani et al.^22^) to 30 years (Zhang et al.^23^ young-adult subgroup), with most cohorts sampling primary or mixed dentition.

Cleft phenotype was heterogeneous. Most studies pooled UCLP, BCLP and isolated CP, while three reported granular composition: Khan et al.^21^ was alveolus-dominant (50%), Hassani et al.^15^ stratified by PNAM exposure, and Shashni et al.^17^ included CLP only. Surgical status at sampling was variably described in four studies and unspecified in six; in Shashni et al.,^17^ 4–9-year-old children spanning pre- and post-palatoplasty stages were not stratified, a gap revisited in the Discussion. Full study-level detail appears in Table 3, Table 4.

### 3.3 Risk of bias assessment

No study reached *Low risk* overall. Five were rated *Some concerns* (Rawashdeh et al.^16^; Sundell et al.^20^; Khan et al.^21^; Sravani et al.^22^; Shi et al.^24^) and five *High risk*. Confounding (D1) dominated, rated *High* in 8 of 10 studies because matching rarely extended beyond age in these small case–control and cross-sectional designs; only Rawashdeh et al.^16^ and Sundell et al.^20^ reached *Some concerns*, through age-stratified and registry-based sampling. In three further studies the overall rating was reasoned down to *Some concerns* because residual confounding was unlikely to reverse the effect, a deviation permitted under ROBINS-E. Post-exposure interventions (D4) were problematic where surgical status was unreported or unstratified, and Funahashi et al.^19^ carried the heaviest profile (*High* on D1, D3, D6 and D7). Exposure measurement (D2) was uniformly *Low*, reflecting standardised culture and 16S rRNA V3–V4 methods. Per-study and per-domain judgements appear in Fig. 2A and B.

### 3.4 Synthesis of results

#### 3.4.1 Oral *Candida* colonisation (MA-1)

Four studies (Rawashdeh et al.^16^; Durhan et al.^18^; Khan et al.^21^; Sravani et al.^22^) contributing 334 participants (CL/P *n* = 171; controls *n* = 163) entered MA-1. Children with CL/P were 2.94 times more likely to harbour oral *Candida* than controls (RR 2.94, 95% CI 2.04 to 4.22; *P* < 0.001), with low heterogeneity (*I*^2^ = 23.4%) and a prediction interval of 1.29 to 6.68. Crude pooled prevalence was 66.1% (113/171) versus 22.1% (36/163). Durhan et al.^18^ contributed data from its post-primary-molar timepoint (11/21 versus 3/13; Fisher exact *P* = 0.075). The remaining studies (Sundell et al.^20^; Funahashi et al.^19^; Hassani et al.^15^; Zhang et al.^23^; Shi et al.^24^) did not provide poolable *Candida* data. Forest and funnel plots are shown in Fig. 3.

**Fig. 3:**
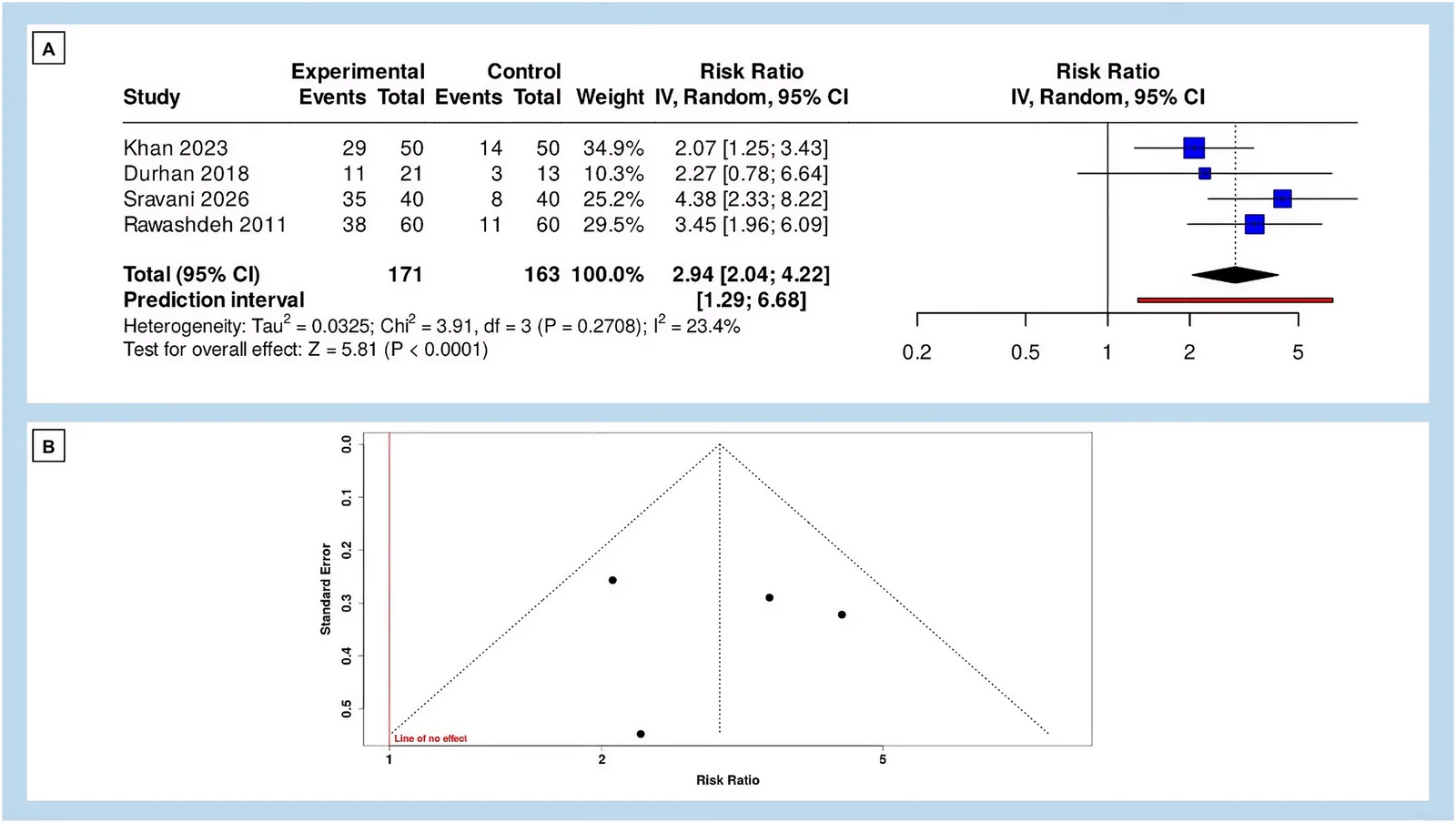
Meta-analysis of oral *Candida* colonisation in children with CL/P versus controls. **(A)** Forest plot (random-effects RR, DerSimonian–Laird); diamond = pooled estimate, RR > 1 favours CL/P. **(B)** Contour-enhanced funnel plot for visual inspection; formal asymmetry tests not performed.

Species-level data showed a parallel compositional shift. *C. albicans* predominated in CL/P (Sravani et al.^22^: 70% versus 15% in controls; Khan et al.^21^: 58% versus 28%, *P* = 0.004), and non-*albicans* species (*C. tropicalis*, *C. krusei*, *C. glabrata*, *C. parapsilosis*) were detected at appreciable frequency (Sravani et al.^22^: 17.5% versus 5%; Khan et al.^21^: multiple species identified).

#### 3.4.2 *Streptococcus mutans* (MA-2)

Three studies (Shashni et al.^17^; Sundell et al.^20^; Durhan et al.^18^) pooling 512 participants (CL/P *n* = 177; controls *n* = 335) showed a 2.74-fold higher risk of high salivary *S. mutans* counts in CL/P (RR 2.74, 95% CI 1.42 to 5.27; *P* = 0.003; 27/177 versus 12/335), with no heterogeneity (*I*^2^ = 0%). The prediction interval (0.65 to 11.51) crossed the null, so the true effect in future settings could plausibly range from modest protection to markedly amplified risk.

Shashni et al.^17^ used a three-arm design in which CL/P (Group I, *n* = 23) was compared against high-caries-risk children (Group II, *n* = 25, selected a priori for deft + DMFT > 5) and caries-free children (Group III, *n* = 25). *S. mutans* counts >10⁶ CFU/mL (Score 3) occurred in 21.7%, 60.0% and 4.0% respectively, differing significantly between Groups I and III (*P* < 0.05) and Groups I and II (*P* < 0.001). To preserve PECO comparability we pooled Group I versus Group III only at the >10⁵ CFU/mL threshold (Score ≥2; 13/23 versus 4/25), excluding the selection-biased Group II. Because the three studies applied different high-count thresholds (≥10^5^ to ≥ 10^6^ CFU/mL; Table 5), this threshold heterogeneity and the intermediate position of CL/P children (4% → 21.7% → 60%) are taken up in the Discussion. Forest and funnel plots are shown in Fig. 4.

**Fig. 4:**
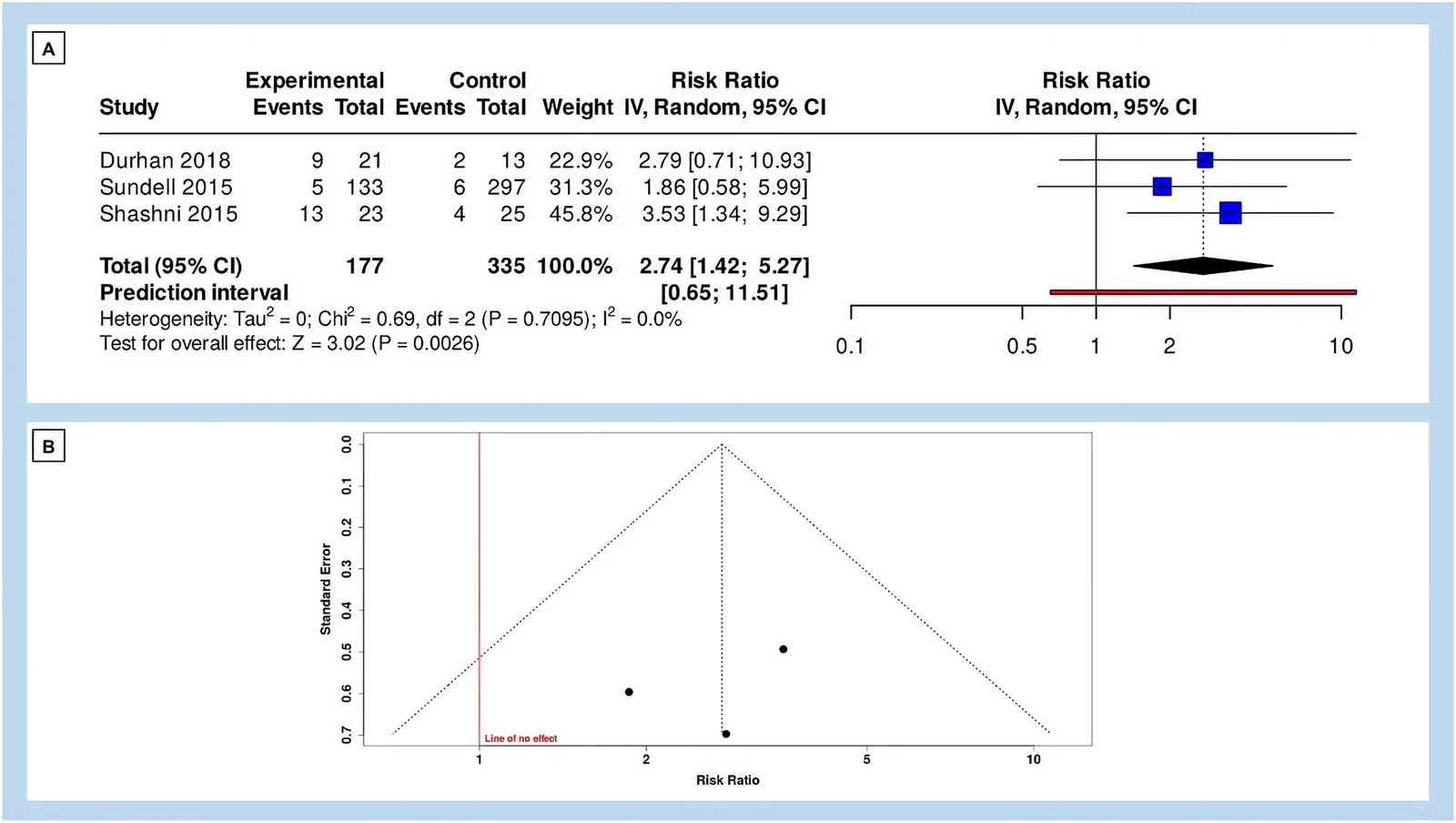
Meta-analysis of high salivary *Streptococcus mutans* carriage in children with CL/P versus controls. **(A)** Forest plot (random-effects RR, DerSimonian–Laird); diamond = pooled estimate, RR > 1 favours CL/P. **(B)** Contour-enhanced funnel plot for visual inspection.

#### 3.4.3 *Lactobacillus* spp. (MA-3)

The same three studies and 512 participants yielded a non-significant pooled estimate (RR 1.33, 95% CI 0.71 to 2.47; *P* = 0.37; 19/177 versus 16/335), with no heterogeneity (*I*^2^ = 0%) and a wide prediction interval (0.34 to 5.19). Here too the studies entered events at thresholds spanning two orders of magnitude (≥10^4^ to ≥ 10^6^ CFU/mL; Table 5).

Two study-level patterns contrasted with this pooled null. At the strict ≥ 10^6^ CFU/mL cut-off feeding MA-3, Sundell et al.^20^ found numerically lower carriage in CL/P (3/133 versus 9/297; RR 0.74), yet reported a highly significant difference across its broader distribution (*P* < 0.001), driven by excess medium-count *Lactobacillus* in CL/P (17% versus 6%); strict thresholds can therefore mask compositional differences. Durhan et al.^18^ likewise detected higher carriage at birth (*P* = 0.029) and at first tooth emergence (*P* = 0.030). This discordance is addressed in the sensitivity analyses and Discussion. Forest and funnel plots are shown in Fig. 5.

**Fig. 5:**
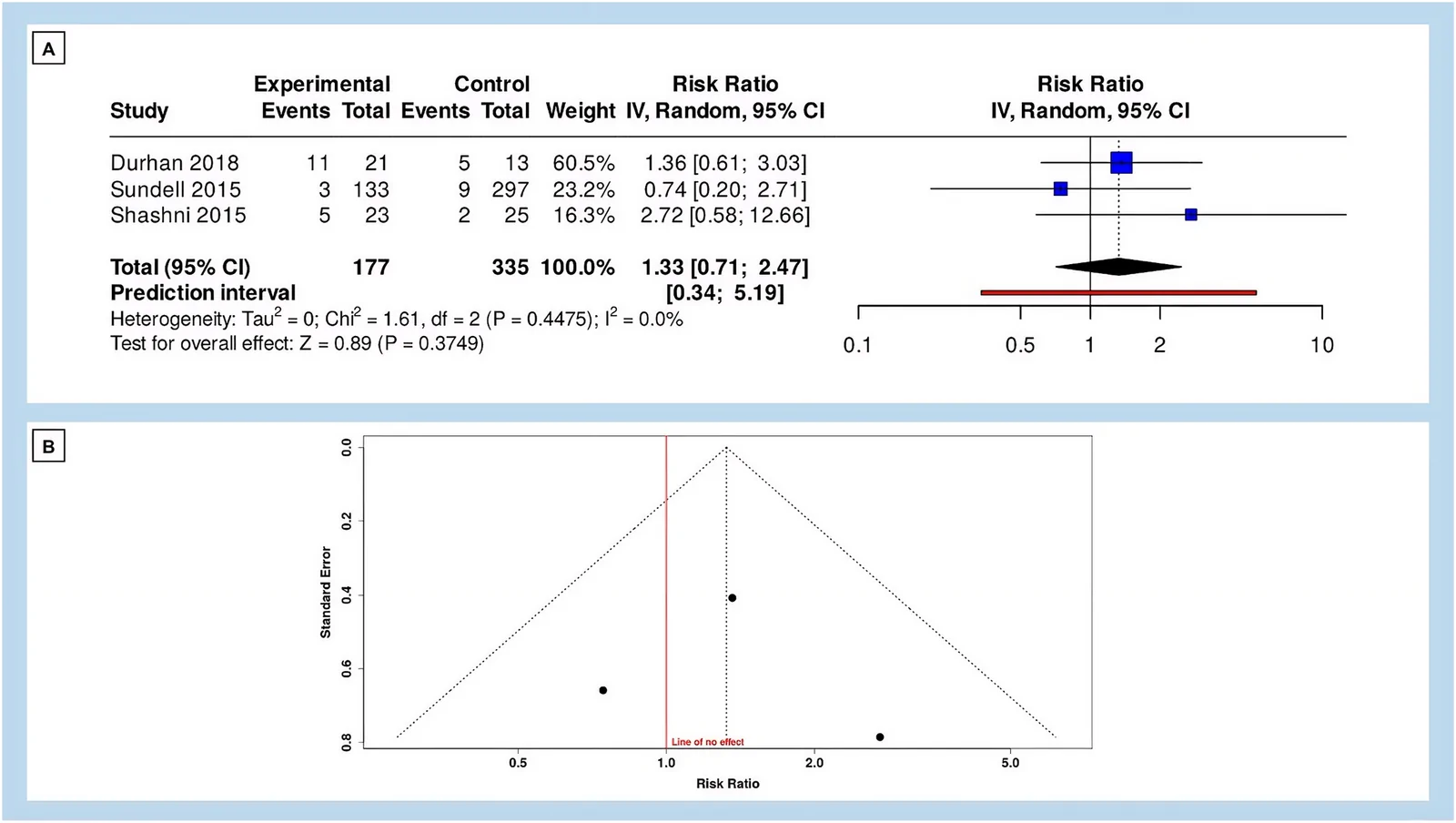
Meta-analysis of high salivary *Lactobacillus* spp. carriage in children with CL/P versus controls. **(A)** Forest plot (random-effects RR, DerSimonian–Laird); diamond = pooled estimate, RR > 1 favours CL/P. **(B)** Contour-enhanced funnel plot for visual inspection.

#### 3.4.4 DMFT/dmft burden (MA-4, narrative)

A pooled SMD across four datasets (Sravani et al.^22^ def; Rawashdeh et al.^16^ DMFT and dmft; Funahashi et al.^19^ DMFT) was 0.74 (95% CI –0.55 to 2.03; *P* = 0.26) with *I*^2^ = 92.3%, and is reported narratively given the very high heterogeneity, which arose from differences in age range, index (def, dmft, DMFT) and sample size (*n* = 6/4 to 60/60). Direction favoured higher caries burden in CL/P in three of four datasets; Funahashi et al.^19^ reversed, reflecting *n* = 6/4 with a single outlier control value (DMFT = 5).

Sravani et al.^22^ (trans-foramen CL/P, 0–3 years, *n* = 40/40) reported def 5.62 ± 0.35 versus 4.83 ± 0.45 (MD +0.79; *P* < 0.001) and a Caries Severity Index (CSI; Chosack^35^) of 2.79 ± 0.33 versus 1.92 ± 0.28 (MD +0.87; *P* < 0.001). Its unusually narrow standard deviations, in a cohort half-composed of 0–6-month-old infants whose deciduous teeth are barely erupted, raise concerns over biological plausibility and yield a disproportionately large effect (SMD ≈ 1.96) that dominates MA-4; a leave-one-out analysis excluding it is reported below.

Rawashdeh et al.^16^ reported DMFT 3.30 ± 2.30 versus 2.50 ± 2.10 (*P* < 0.001) and dmft 2.93 ± 2.10 versus 1.97 ± 1.40 (*P* = 0.015; *n* = 60/60), and Funahashi et al.^19^ DMFT 0.67 ± 0.82 versus 1.25 ± 2.50 (*n* = 6/4). Shashni et al.^17^ reported deft 8.65 ± 5.80 versus 12.64 ± 3.43 (*P* < 0.01) and deft + DMFT 10.14 ± 6.09 versus 12.73 ± 3.42 (*P* = 0.149) against Group II; these selection-biased benchmarks were excluded a priori from MA-4.

#### 3.4.5 Caries prevalence (MA-5, single-study)

Durhan et al.^18^ was the only study reporting caries prevalence as a binary outcome. At first primary-incisor eruption (≈8–15 months), 4/21 CL/P infants (19.0%) versus 0/13 controls (0%) showed initial caries by ICDAS-equivalent criteria (Fisher exact *P* = 0.144); at the post-primary-molar timepoint (≈24–30 months), 10/21 (47.6%) versus 5/13 (38.5%) were affected (*P* = 0.728). The first-incisor timepoint is taken as the primary estimate given its clearer separation and pre-specified timing, with the later timepoint retained for transparency and revisited in the sensitivity analyses.

#### 3.4.6 Sensitivity analyses

For MA-1, sequential leave-one-out removal yielded RR estimates between 2.41 (95% CI 1.62 to 3.59) and 3.43 (95% CI 2.31 to 5.10), all retaining significance. For MA-2, excluding Shashni et al.,^17^ the heaviest contributor (45.8% weight, highest RR 3.53), shifted the pooled RR from 2.74 to 2.18 (95% CI 1.05 to 4.52) while preserving significance, so no single study drove the result. For MA-3, the estimate stayed non-significant throughout; this persistent null contrasts with significant study-level differences in Sundell et al.^20^ (*P* < 0.001) and Durhan et al.^18^ (at birth *P* = 0.029, at first tooth emergence *P* = 0.030) measured at other thresholds, indicating threshold-driven divergence rather than a single outlier.

For MA-4, a pre-specified leave-one-out excluding Sravani et al.,^22^ prompted by the plausibility concerns above, attenuated the pooled SMD to +0.42 (95% CI 0.17 to 0.67) and collapsed the heterogeneity (*I*^2^ = 0%; *P* ≈ 0.001). Sravani thus drove both the extreme magnitude and the very high heterogeneity, although the underlying direction, higher caries burden in CL/P, held at a much smaller effect size across the remaining datasets.

For the single-study MA-5, adopting the post-primary-molar timepoint (47.6% versus 38.5%; *P* = 0.728) in place of the first-incisor timepoint would attenuate the between-group separation and yield a non-significant estimate; the first-incisor timepoint was retained as primary for the reasons set out above.

Egger's regression for small-study effects was not undertaken because each meta-analysis comprised fewer than ten studies (Cochrane Handbook Section 13.3.5.5; https://training.cochrane.org/handbook). Pre-specified subgroup analyses by cleft phenotype, surgical status and geographical region were also not performed, as no stratum reached the a priori minimum of four studies.^36^

#### 3.4.7 Certainty of evidence (GRADE)

Applying GRADE, MA-1 was rated Low (serious risk of bias, predominantly D1, plus moderate imprecision).^34^ MA-2 was rated Very Low (serious risk of bias; serious indirectness from heterogeneous high-count thresholds, ≥ 10^5^ versus ≥ 10^6^ CFU/mL, and the non-representative control selection in Shashni et al.^17^; moderate imprecision). MA-3 was Very Low (serious risk of bias; serious imprecision with the CI crossing the null), as were MA-4 (serious inconsistency, *I*^2^ = 92.3%) and the single-study MA-5 (very serious imprecision). The Summary of Findings is given in Table 6.

## 4 Discussion

### 4.1 Principal findings

This synthesis quantified three cariogenic microorganisms and four caries-experience indices in CL/P. Oral *Candida* carriage was nearly threefold higher than in controls (RR 2.94, 95% CI 2.04 to 4.22; *I*^2^ = 23.4%), with a prediction interval above unity (1.29 to 6.68) that points to a reproducible over-representation. High salivary *S. mutans* counts were of similar size (RR 2.74, 95% CI 1.42 to 5.27), though a prediction interval crossing unity (0.65 to 11.51) helped pull GRADE to Very Low. *Lactobacillus* showed no pooled difference at the strict MA-3 thresholds, a divergence we read as threshold-driven rather than biological. Caries indices favoured CL/P in three of four datasets, but very high heterogeneity (*I*^2^ = 92.3%) precluded a firm pooled estimate. Overall, the evidence supports an early-life fungal–bacterial shift whose caries consequences remain uncertain in magnitude.

### 4.2 Interpretation of oral *Candida* burden in CL/P

Our pooled estimate, a 2.94-fold higher *Candida* risk in CL/P (95% CI 2.04–4.22; *I*^2^ = 23.4%; *k* = 4; *n* = 334; prediction interval 1.29–6.68), holds its direction across plausible future settings. Its closest comparator, Souza et al.^9^, reported colonisation in 89.5% of infants aged 0–18 months with cleft palate, 100% with acrylic occlusal plates and 44% in controls (*p* = 0.037), with *C. albicans* dominant and multi-species co-colonisation in 30.5% of positive cultures. That study shows *Candida* is already elevated in the first months of life, before any appliance is fitted.

A second anchor, Rawashdeh et al.,^16^ found carriage of 63.3% versus 18.3% in a Jordanian cohort aged 5–17 years. That 45-percentage-point gap sits nearer the centre of our pooled risk ratio than the gap in Souza et al.,^9^ implying that age, phenotype and appliances shift absolute prevalence while the relative risk holds steady.

Species data converge on *C. albicans* as dominant across ages: Pinto-Almazán et al.^37^ found oral *Candida* in 6.99% of 743 Mexican children aged 3–6 years, with *C. albicans* 87.8% of isolates. This may carry clinical relevance, given the broad virulence potential generally attributed to *C. albicans* in the oral cavity.

The acrylic-plate signal in Souza et al.^9^ foreshadows the biofilm question addressed below. Linking carriage to cariogenic risk, Man et al.^38^ quantify the *Candida*–biofilm coupling in early childhood caries (plaque OR 7.98, 95% CI 3.84–16.58, *I*^2^ = 84%; saliva OR 9.42, 95% CI 4.15–21.40, *I*^2^ = 50%).

### 4.3 Linking oral *Candida* to cariogenic burden in CL/P

Does this fungal over-representation translate into more caries? The most direct answer comes from Escobar-Arregocés et al.,^5^ who pooled five of twenty-three eligible studies in non-syndromic CL/P and found an odds ratio of 2.03 for caries experience versus peers (*p* < 0.005). Although 90% of those studies were at high risk of bias, the direction and size match our MA-2 *S. mutans* estimate (RR 2.74, 95% CI 1.42–5.27), placing the *Candida* signal within a coherent cariogenic story rather than isolated colonisation.

Mechanistic support comes from recent *in vitro* work. Sansanoa et al.^11^ showed that dual-species biofilms of *S. mutans* with *C. albicans*, *C. glabrata* or *C. tropicalis* built more mass than the sum of their mono-species controls (*p* < 0.05), fell from pH 7 to about 4.5 within 2 h, and stayed below the critical demineralisation threshold of 5.5 for the full 36 h; *gtfB* and *gtfD* were upregulated across most pairings, with *C. tropicalis* the most cariogenic. Jin et al.^39^ cast this as a carbohydrate-metabolism partnership in which GtfB binds the *Candida* mannan layer and supplies glucan substrate, while Francis et al.^10^ show that it also recruits Als1 and Hwp1 adhesins to anchor the bacterium to fungal cells, yielding a polymicrobial biofilm with enhanced acid output.

The convergence of the epidemiology Escobar-Arregocés et al.^5^ with the biofilm evidence (Sansanoa et al.^11^) supports a *Candida*-mediated cariogenic pathway in CL/P, though CL/P populations remain scarce in the *in vitro* literature and causality cannot be drawn from our data. The downstream steps, co-aggregation and cleft-specific ecological plaque shifts, are taken up below.

### 4.4 Cariogenic shift mechanism in CL/P

This *Candida*–bacterial co-occurrence fits the ecological plaque hypothesis, in which sustained acidic, nutrient-stressed conditions select for acidogenic, aciduric species and erode diversity.^40^ CL/P-specific data bear this out: across four caries states, Ace and Chao indices differed (*p* = 0.013), the Microbial Dysbiosis Index exceeded controls (*p* = 0.004), *Streptococcus*, *Gemella*, *Actinomyces*, *Scardovia* and *Olsenella* were enriched in high-caries CL/P, the facultatively anaerobic phenotype rose (*p* = 0.038), and *Selenomonas* acted synergistically with *S. mutans* to worsen severity.^24^ Alveolar-cleft plaque followed suit, enriched for *Neisseria*, *Haemophilus*, *Aggregatibacter* and *Porphyromonas* with raised potentially pathogenic, Gram-negative phenotypes.^23^

Molecularly, *S. mutans* virulence here runs on glucan synthesis via *gtfB/C/D* and the *vicRK* and *liaSR* regulators that coordinate adhesion, EPS-matrix assembly and acid-stress survival.^41^ Glucan is 10–30% of biofilm dry weight, and a two-step expansion (osmotic spread of glucan-rich EPS, then colony formation on hydroxyapatite) drives lateral spread, with biofilm centres acidifying to pH 5.7 and new colonies failing without hydroxyapatite.^42^ *Lactobacillus* binds sound enamel only weakly, but adheres readily once *S. mutans* has built retention sites, after which *L. plantarum*, *L. salivarius*, *L. rhamnosus* and *L. casei* can drop pH to 5.5 within 2.5–3 h and sustain demineralisation.^43^

In CL/P the post-surgical anatomy amplifies all of this: even after fistula closure, residual irregularities and altered salivary clearance keep the community tilted towards potentially pathogenic taxa, with retention sites favouring the lineages most tied to caries.^24^

### 4.5 Clinical implications

The pairing of cariogenic bacterial enrichment with high *Candida* carriage, shown here and backed by mechanistic and microbiome data, argues that prevention in CL/P should target both the bacterial and fungal arms of the microbiota and begin before structural caries appear.^9^^,^^24^

Topical 5% sodium fluoride varnish remains the best-supported preventive measure. A Community Guide review of 31 studies (60,780 students) found school varnish programmes cut caries initiation by a median 32% (IQI 21–37%) in permanent and 25% (IQI 4–37%) in primary teeth, including in high-risk and low-socioeconomic groups.^44^ Where moisture control or cooperation is poor, both common in CL/P, a single glass-ionomer sealant on primary second molars matched quarterly varnish for caries-into-dentin at 12 months (7.8% vs 8.0%, *p* = 0.913), though sealant retention fell to 13.5%.^45^ Adjunctive probiotics (*Lactobacillus* and *Bifidobacterium*) lower salivary *S. mutans* (MD −0.40 CFU/mL, 95% CI −0.57 to −0.24; moderate certainty, 19 RCTs), needing at least six months of continuous intake.^46^

Recall intervals should therefore be shorter than the population norm, periodic screening should include assessment for *Candida* carriage in symptomatic children, and caregiver education ought to be embedded within the multidisciplinary CL/P care pathway from the deciduous-dentition stage onwards.

### 4.6 Limitations and heterogeneity

Several limitations temper these conclusions. First, clinical heterogeneity was substantial: participants spanned a wide age range, several non-syndromic cleft phenotypes (cleft lip only, cleft palate only, and combined cleft lip and palate) and differing surgical and orthodontic stages, each independently associated with oral microbial composition and caries risk. Pre-specified subgroup analyses could not be performed because no stratum reached the minimum of four studies, so effect modification could not be explored.

Second, methodological heterogeneity in microbial detection was pronounced. Studies used selective culture on MSA or CHROMagar, qPCR for species-specific loci, and 16S rRNA or ITS amplicon sequencing, modalities that quantify different fractions of the oral community and are not directly interchangeable, so residual measurement bias cannot be excluded despite our restriction of pooled estimates to comparable outcomes. Caries scoring also varied, despite harmonisation to Chosack^35^ dmfs/DMFS criteria where re-extraction was possible.

Third, the evidence base was dominated by cross-sectional and case–control work, with only one prospective design, which precludes causal inference between fungal–bacterial co-carriage and incident caries and limits insight into temporal dynamics across the surgical–orthodontic timeline.

Fourth, most studies raised concerns over confounder adjustment, particularly oral hygiene, dietary sugar and socioeconomic status, variables that independently shape both microbial carriage and dmfs/DMFS. Because every synthesis drew on fewer than ten studies, formal funnel-plot asymmetry tests such as Egger's regression were not undertaken (see the sensitivity analyses); small-study effects and publication bias therefore cannot be excluded, although leave-one-out analyses left each pooled estimate materially unchanged.^36^

Finally, although no language or date restrictions were applied, the exclusion of grey literature may still have introduced publication bias, and the concentration of studies in Asian and European centres limits generalisability to African and Latin American CL/P populations, where access to early surgical repair and preventive care differs substantially.

### 4.7 Implications for future research

These limitations map onto clear research priorities. Prospective cohorts that follow CL/P children from primary through mixed dentition, with standardised microbial sampling that combines culture-based quantification and shotgun metagenomics, would clarify whether *Candida*–streptococcal co-carriage precedes or follows caries onset. Pragmatic randomised trials of preventive interventions (fluoride varnish, glass-ionomer sealants and targeted probiotic adjuncts) should be designed specifically for CL/P anatomy, with caries increment and microbial endpoints reported jointly. Individual-participant-data syntheses would enable adjustment for the oral hygiene, dietary and socioeconomic confounders that aggregate-data meta-analyses cannot resolve, and inclusion of cohorts from low- and middle-income settings is essential to test the external validity of the burden documented here.

## 5 Conclusion

Within the limits of observational evidence, children and adolescents with cleft lip and/or palate carried a higher oral *Candida* burden (RR 2.94, 95% CI 2.04–4.22) and more frequent high salivary *Streptococcus mutans* counts (RR 2.74, 95% CI 1.42–5.27) than non-cleft peers, whereas *Lactobacillus* differences were threshold-dependent and non-significant. Caries indices were directionally higher in three of four datasets, but very high heterogeneity precluded a stable pooled estimate, and GRADE certainty was Low to Very Low. These findings support integrating *Candida* assessment into routine caries-risk evaluation from the deciduous-dentition stage, alongside professionally applied fluoride and structured preventive care within the multidisciplinary cleft pathway.

## Consent to participate

Not applicable. No participants were recruited for this review; consent was governed by the procedures of each primary study.

## Statement of informed consent

No contact with patients or their parents or guardians occurred. Data requested from primary-study authors were limited to group-level summary statistics, so no further consent was required.

## Author contributions (CRediT)

M.I.H. and E.A.A. contributed equally to this work and are designated joint first authors. M.I.H. and A.T. conceived and designed the review. M.I.H. and E.A.A. developed the search strategy, screened records, extracted data, and appraised risk of bias. M.I.H. performed the statistical synthesis and drafted the manuscript. I.T. was pre-designated as adjudicator for study selection and appraisal. H.M., I.P.A., I.T., and A.T. provided supervision and senior clinical interpretation. A.T., as corresponding author, coordinated the project and oversaw its submission. All authors critically revised the manuscript and approved the final version for submission.

## Ethics approval

Not required. This review analysed previously published data and collected no new data from humans or animals.

## Consent for publication

Not applicable. Only aggregated, study-level data are presented; no individual data, images or identifiable details are included.

## Data availability

All data analysed in this review are available within the cited primary studies and the accompanying tables; no new datasets were generated during the current study.

## Protocol registration

The review protocol was prospectively registered with the International Prospective Register of Systematic Reviews (PROSPERO) under registration number CRD420261402624.

## Statement of human and animal rights

All data came from published primary studies, assumed to have been conducted in accordance with the ethical standards of their institutional and national research committees and the 1964 Declaration of Helsinki and its later amendments.

## Funding

This research received no specific grant from any funding agency in the public, commercial, or not-for-profit sectors.

## Declaration of competing interest

The authors declare that they have no known competing financial interests or personal relationships that could have appeared to influence the work reported in this paper.
